# Acute and Long-Term Effects of Hyperthermia in B16-F10 Melanoma Cells

**DOI:** 10.1371/journal.pone.0035489

**Published:** 2012-04-20

**Authors:** Mónica Pereira Garcia, José Roberto Tinoco Cavalheiro, Maria Helena Fernandes

**Affiliations:** 1 Laboratório de Farmacologia e Biocompatibilidade Celular, Faculdade de Medicina Dentária, Universidade do Porto (FMDUP), Porto, Portugal; 2 Instituto de Engenharia Biomédica (INEB), Universidade do Porto, Porto, Portugal; University of Tennessee, United States of America

## Abstract

**Objective:**

Hyperthermia uses exogenous heat induction as a cancer therapy. This work addresses the acute and long-term effects of hyperthermia in the highly metastatic melanoma cell line B16-F10.

**Materials and Methods:**

Melanoma cells were submitted to one heat treatment, 45°C for 30 min, and thereafter were kept at 37°C for an additional period of 14 days. Cultures maintained at 37°C were used as control. Cultures were assessed for the heat shock reaction.

**Results:**

Immediately after the heat shock, cells began a process of fast degradation, and, in the first 24 h, cultures showed decreased viability, alterations in cell morphology and F-actin cytoskeleton organization, significant reduction in the number of adherent cells, most of them in a process of late apoptosis, and an altered gene expression profile. A follow-up of two weeks after heat exposure showed that viability and number of adherent cells remained very low, with a high percentage of early apoptotic cells. Still, heat-treated cultures maintained a low but relatively constant population of cells in S and G_2_/M phases for a long period after heat exposure, evidencing the presence of metabolically active cells.

**Conclusion:**

The melanoma cell line B16-F10 is susceptible to one hyperthermia treatment at 45°C, with significant induced acute and long-term effects. However, a low but apparently stable percentage of metabolically active cells survived long after heat exposure.

## Introduction

Hyperthermia, a cancer therapy, uses exogenous heat induction as therapeutic agent [1]. It can be used alone or as adjuvant to other therapies, especially chemotherapy and radiotherapy [2]. Hyperthermia localized techniques apply heat only to the tumour using electromagnetic energy ultrasounds [3]. The use of hyperthermia is controversial and presents significant technical challenges. Scientific reports show contradictory results relatively to hyperthermia therapeutic potential, some attesting its utility and others showing no effect. This is probably due to the lack of knowledge correlating the complex hyperthermic effects at cellular and tissue level [1].

*In vivo* animal studies have been performed in a variety of tumour models, as melanoma [4], glioma [5], mammary carcinoma [6], osteosarcoma [7], prostate cancer [8], most showing some benefit with the use of hyperthermia. These studies have shown that with hyperthermia exposure involving lower temperatures, as 43°C, the tumour regression can only be observed with more than one treatment session [5], [7], [9], [10]. In this situation, it has been reported that the hyperthermia treatment gives rise to an immune reaction against the tumour cells, leading to a second deleterious effect to the tumour, which appears to be important to a good outcome [11], [12]. On the other hand, higher temperatures, as 45–46°C, lead to tumour regression with fewer applications; in this case, the heat shock reaction takes place immediately, and tumour treatment is faster, apparently without side effects [8], [11].

*In vitro* studies have demonstrated a variety of effects, for heating temperatures ranging from 41–46°C and different heating times. At cellular level, the heat shock effect involves almost all known structural and functional systems, namely alterations in gene expression and ion homeostasis, protein degradation and denaturation, which can be reversible or lethal [1], [2], [13], [14], [15], [16], [17], [18]. Depending on the treatment protocol, hyperthermia outcome alternates from cells that resist to damage and are able to recover, to cells that can not compensate and undergoes predominantly apoptosis, to cells that immediately suffer necrosis [17]. A variety of results have been reported, depending on the cell line, temperature and exposure time [17], [19], [20], [21], [22]. Regarding melanoma cells, significant differences in the experimental protocols have been reported, namely involving human melanoma cells heated at temperatures ranging from 41–43°C for 3–6 h [14], 41.8–45°C for 15–150 min [15] and 43°C for 120 min [19] and, also, B16-F10 murine melanoma cells heated at 43 and 46°C for 30 min [23]. Differences were also observed in the characterization of the heat shock reaction, but these studies have focused in the acute effects of the thermal treatment, i.e. evaluated immediately after the heat shock. However, independently of the treatment protocol (temperature and exposure time), there are always the possibility of surviving cells, whose long-term behaviour has received little attention.

In this context, the aim of this work was to study the acute and long-term effects of hyperthermia, 45°C for 30 min, in the highly metastatic murine melanoma cell line B16-F10. The heat shock reaction was characterized by testing viability/proliferation, F-actin cytoskeleton organization, mechanism of cell death, cell cycle analysis and expression of functional melanoma genes and genes associated with the heat shock reaction.

## Materials and Methods

### Cell culture experiments

B16-F10 cell line from C57BL/6J mice was purchased from ATCC. Cells were cultured in 90 mm culture plates and were maintained in standard culture conditions, i.e., α-minimal essential medium (α-MEM) containing 10% foetal bovine serum (FBS), 2.5 µg/ml fungizone, penicillin-steptomycin (100 IU/ml and 10 mg/ml, respectively), and incubated in a humidified atmosphere of 5% CO_2_ in air at 37°C. Culture medium was changed twice a week. At 70 to 80% cell confluence, adherent cells were enzymatically released with a solution of 0.04% trypsin in 0.25% EDTA.

B16-F10 cells, seeded at 5×10^3^ cell/cm^2^, were cultured in the experimental conditions described above. At day 5, 50–60% confluence, cell cultures were submitted to a hyperthermia treatment. In this procedure, culture plates, sealed with parafilm sealing film, were totally submerged in a water bath and incubated at 45°C (±0.1°C) for 30 minutes. Following, the heat-treated cultures were kept for a further 14 days at 37°C, and the medium was changed twice a week. Control cultures, which were kept at 37°C, were evaluated simultaneously.

Control and heat-treated cultures were characterized immediately after the treatment (0 h) and throughout the further 14 d after the heat shock, as follows.

### Cell viability/proliferation

B16-F10 melanoma cells were cultured (5×10^3^ cell/cm^2^) in 96-well culture plates in standard culture conditions. Cell viability/proliferation of control and heat-treated cultures was analysed by the MTT and LDH assays.

The MTT (3-(4,5-Dimethylthiazol-2-yl)-2,5-diphenyltetrazolium) assay is a method based in the capability of mitochondrial dehydrogenase to transform the MTT in a dark blue formazan product [24], [25]. MTT (0.5 mg/ml) was added to each well, and the culture plates were incubated for 2 h at 37°C. Following, the formazan salts were dissolved with 100 µl of dimethylsulphoxide (DMSO) and the absorbance (Abs) was determined at λ = 600 nm on an Elisa reader (Synergy HT, Biotek).

The lactate dehydrogenase (LDH) assay is based on the reduction of NAD by the action of LDH released to the medium [26]. The resulting reduced NAD (NADH) is utilized in the stoichiometric conversion of a tetrazolium dye. Determination of the total LDH was performed in control and heated culture plates using the In vitro toxicology assay kit lactate dehydrogenase based (Sigma-Aldrich; St. Louis, MO). To determine the total LDH in the culture, LDH assay lysis solution (1/10 of culture medium volume) was added to the wells and the plates were incubated at 37°C for 45 minutes. Medium was centrifuged at 250 g for 4 minutes to pellet debris. Samples aliquots (25 µl) were mixed with 50 µl lactate dehydrogenase assay mixture (LDH assay substrate, cofactor and dye solution), and incubated for 20 minutes at room temperature in the dark. Absorbance was determined at λ = 492 nm on a Elisa reader (Synergy HT, Biotek). The amount of LDH leakage to the medium was normalized by total LDH, and calculated as follows: LDH leakage % =  LDH medium/total LDH ×100.

### Cell morphology and F-actin cytoskeleton labelling

B16-F10 melanoma cells were cultured in 30 mm culture plates (5×10^3^ cell/cm^2^) in standard culture conditions. Control and heat-treated cultures were fixed at defined time points with formaldehyde 3.7% (methanol free). After, cells were labelled for F-actin cytoskeleton and nucleus and observed by confocal laser scanning microscopy (CLSM).

For labelling, cells were permeabilized with 0.1% triton in PBS for 5 min. After, cultures were incubated with 10 mg/mL of BSA with 1 µg/mL RNAse in PBS, for 1 h. Following, they were treated with 5 U/mL Alexa Fluor® 488-Phalloidin (Invitrogen, Barcelona, Spain), for 20 min in the dark. Cells were washed with PBS and a solution of propidium iodide (PI,10 µg/mL in PBS; Sigma-Aldrich, St. Louis, MO) was added (10 min in the dark, at room temperature). Cells were maintained in mounting medium (20 mM Tris pH 8.0, 0.5% N-propyl gallate, 90% Glycerol) before CLSM observation. CLSM was performed in a Laser Scanning Confocal Microscope Leica SP2 AOBS SE (Leica Microsystems, Germany).

### Mechanism of cell death and cell cycle analysis

B16-F10 melanoma cells were cultured (5×10^3^ cell/cm^2^) in 75 cm^2^ culture flasks, in the conditions described above. At defined time-points, control and heat-treated cell layers were washed twice in PBS and cells were enzymatically released (0.04% trypsin in 0.25% EDTA). The resulting cell suspension, reflecting the number of adherent cells in control and heat-treated cultures, was used to evaluate the mechanism of cell death and cell cycle phase.

#### Mechanism of cell death

Alterations in the apoptotic cell index, namely the relative percentage of cells undergoing early and late apoptosis, were assessed with TACS^TM^ Annexin V-FITC Apoptosis Detection Kit (R & D Systems) that includes Annexin V conjugated to FITC and PI. Early in the apoptotic process, caspases and other proteases cleave the cytoskeleton and the plasma membrane loses its rigidity, due to the deleterious effects in the underlying net of actin. This process disrupt the phospholipid asymmetry, leading to an increase of exposure of phosphatidylserine (PS) on the outer leaflet of plasma membrane [27]. Annexin V binds to PS; still, at this stage, damaged plasma membrane is ion selective for a long time excluding PI (Annexin V-FITC^+^/PI^−^; early apoptotic cells). In later stages of apoptosis, cells enter a blebbing process, leading to a shrunken morphology, and loose the membrane integrity and the ability to exclude PI [27], [28] (Annexin V-FITC^+^/PI^+^; late apoptotic and/or necrotic cells). Normal, viable cells are unstained. In the experimental protocol used, the cell suspension obtained as described above was centrifuged at 500 g for 7 min. Cells were re-suspended in PBS with 1% of BSA, and an aliquot of 10^6^ cell/ml was used for apoptotic cell index determination. After a centrifugation at 500 g for 7 min, cell pellets were incubated with 50 µL Annexin V incubation reagent (5 µL 10× binding buffer, 5 µL PI, 0.5 µL Annexin V-FITC, 39.5 µL dH_2_O), for 20 min in the dark, at room temperature. After this period, 500 µL of 1× Binding buffer was added, and the samples were assessed by flow cytometry analysis.

#### Cell cycle analysis

Alterations in the cell cycle were assessed with PI. An aliquot of 10^6^ cell/mL, from the cell suspension obtained as described above, was fixed with formaldehyde 1.9% (methanol free) in PBS and kept at 4°C. Fixed cells were washed twice in PBS, and centrifuged at 500 g for 7 min. Cells were stained in 1 mL PBS, 50 µg/mL PI and 50 µg/mL RNAse (1 h, in the dark at room temperature), and assessed for cell cycle analysis, by flow cytometry.

### Total RNA extraction and RT-PCR analysis

Control and heat-treated cultures of B16-F10 melanoma cells were analysed by RT-PCR, during the first 24 h following the heat treatment, to access the expression of the housekeeping gene β-actin, TYR (Tyrosinase), TRP-1 (Tyrosinase related protein 1), TRP-2 (Tyrosinase related protein 2), pMel17 (Premelanosome protein), MITF (Microphthalmia-associated transcription factor) and MART-1 (Melanoma associated antigen recognized by T-cells 1), Bcl-2, BAX, p53 and Hsp70 (Heat shock protein 70). Total RNA was extracted using the Nucleospin RNA II kit (Machery-Nagel), according to the manufacturer's instructions. RNA was analysed for its concentration and purity in each sample by UV spectrophotometry at 260 nm and by calculating the A_260nm_/A_280nm_ ratio, respectively. RT-PCR was done using the Titan One Tube RT-PCR System (Roche Applied Science), according to the manufacturer's instructions. Half microgram of total RNA from each sample was reverse transcribed and amplified (25 cycles) with Titan One Tube RT-PCR System (Roche), with an annealing temperature of 50°C. The amplification conditions were chosen accordingly to preliminary calibration experiments (data not shown), in order to become possible to use the RT-PCR analysis as a quantitative tool. The primers used (Sigma Genosys) are listed on Table 1. RT-PCR products were separated on a 1% (w/V) agarose gel and gel band intensities were analysed with ImageJ 1.41 software. Values were considered as a percentage of the corresponding β-actin value of each experimental condition.

**Table 1:** Primers used on RT-PCR analysis.

Gene	5′ Primer	3′ Primer
β-actin	AAGAGCTATGAGCTGCCTGA	CAGGAGGAGCAATGATCTTG
TYR	GGCCAGCTTTCAGGCAGAGGT	TGGTGCTTCATGGGCAAAATC
TRP-1	GCTGCAGGAGCCTTCTTTCTC	AAGACGCTGCACTGCTGGTCT
TRP-2	GGATGACCGTGAGCAATGGCC	CGGTTGTGACCAATGGGTGCC
pMel17	CGGATGGTCAGGTTATCTGG	ATGGTGAAGGTTGAACTGGC
MITF	GTATGAACACGCACTCTCTCGA	CTTCTGCGCTCATACTGCTC
MART-1	TGCCCCAAGAAGACATTCAC	GTGAATAAGGTGGCGGTGAA
Bcl-2	CTCGTCGCTACCGTCGTGACTTCG	CAGATGCCGGTTCAGGTACTCAGTC
BAX	AAGCTGAGCGAGTGTCTCCGGCG	GCCACAAAGATGGTCACTGTCTGCC
p53	ATGACTGCCATGGAGGAGT	CTCGGGTGGCTCATAAGGTA
Hsp70	CCATCCAGAGACAAGCGAAG	CGTTTAGACCGCCGATCACA

### Statistical analysis

Four independent experiments were performed. In the cell viability/proliferation assays (MTT and LDH assays) four replicas were performed in each experiment. Quantitative data are presented as mean ±SEM. Data were analysed with Mann Whitney U test. A p value of ≤0.05 was considered statistically significant.

## Results

### Cell viability/proliferation

**Figure 1:**
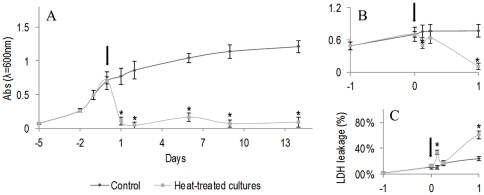
Cell viability/proliferation of B16-F10 melanoma cells submitted to a heat treatment (HT), 45°C for 30 min. Cultures were heated at day 5 (Arrow) and further maintained at 37°C for 2 weeks. A: MTT assay, throughout the culture period; B: Detail of the MTT assay during the first 24 h after the heat treatment; C: LDH assay during the first 24 h after the heat treatment. *Significantly different from control (cultures kept at 37°C).

Figure 1A shows the results of the MTT assay. In control cultures, kept at 37°C, cell viability/proliferation increased gradually with the culture time, especially during the first week; afterwards, cultures reached confluence, and the MTT reduction values increased slowly. The hyperthermia treatment, performed at day 5 of the culture, resulted in a significant decrease in the viability/proliferation during the first 24 hours after the heat shock, Figure 1A. Within this period, the cell response was not uniform, i.e. there was a slight decrease in the viability during the first 3 h, followed by a partial recovery (found at 6 h), but, afterwards, cell viability decreased significantly, Figure 1B; 24 h after the heat treatment, values were in average 80% lower than those observed in control cultures, Figure 1B. Following, 2 d to 14 d after the heat treatment, cell viability/proliferation remained very low, compared to the control cultures, Figure 1A.

Results from the LDH assay were in line with those found in MTT assay. Figure 1C shows the results observed in the first 24 h following the heat treatment. In control cultures, LDH leakage remained low, but heat treated cultures showed an increase in the amount of LDH released to the medium 3 h after the treatment, followed by a decrease after 6 h, and, again, an increase in the next hours.

### Cell morphology and F-actin cytoskeleton organization

**Figure 2:**
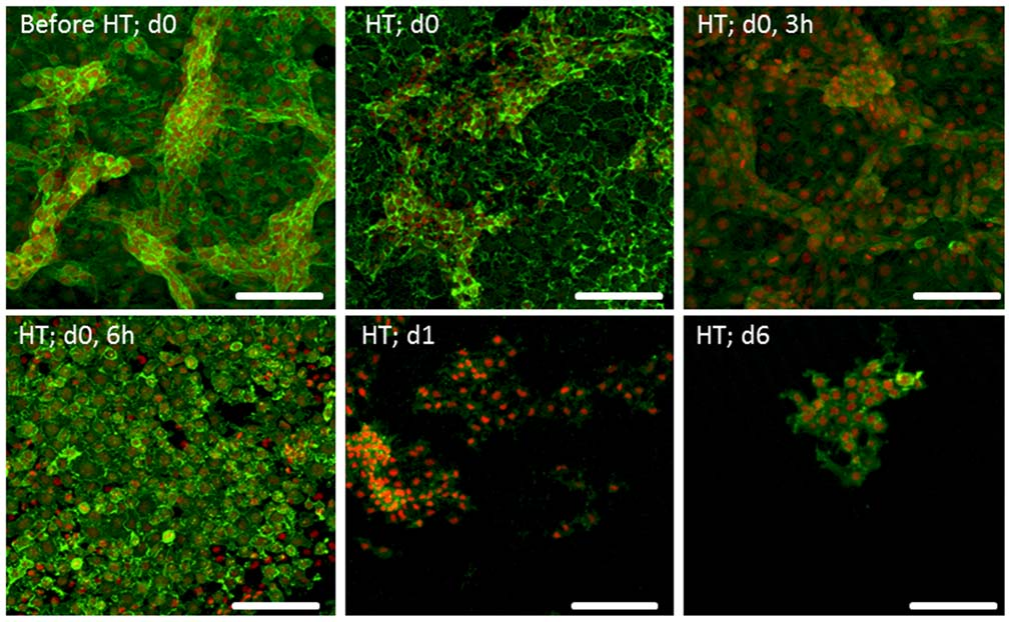
CLSM observation of B16-F10 melanoma cells submitted to a heat treatment (HT), 45°C for 30 min, stained for F-actin cytoskeleton (green) and nucleus (red). Representative images (from four independent experiments) showing the effect of heat exposure on cell morphology and cell layer organization, at defined time-points (d 0, 0 h–d 6, immediately to 6 days after heat exposure). Bar represents 400 µm.

Control and heat-treated cultures were stained for the F-actin cytoskeleton and nucleus and representative CLSM images are shown in Figure 2.

Control cultures presented an organized cell layer with extensive cell-to-cell contact and areas of higher cell density. In addition, cells displayed a spread F-actin cytoskeleton with well-defined peripheral limits and a prominent nucleus. The heat treatment induced significant alterations on this behaviour. Deleterious effects were observed immediately after the heat shock (time point, 0 h) and were well evident 3 h after the treatment, reflected mainly by a lower definition of the dense peripheral F-actin ring. After 6 h, some recovery in the organization of the cytoskeleton occurred, as cells displayed a better definition of the peripheral limits; still, cultures presented alterations in the organization of the cell layer and the presence of some rounded and shrunken cells. However, 1 d after the heat shock, cultures appeared greatly affected, with evident alterations in the F-actin cytoskeleton, loss of the cell limits, rounded shape, cytoplasm shrinkage and condensation of the nuclear material. Other effects included loss of the organization of the cell layer and cell-to-cell contact and a significant decrease in the number of attached cells. These deleterious effects were observed throughout the culture time.

### Mechanism of cell death and cell cycle analysis

**Figure 3:**
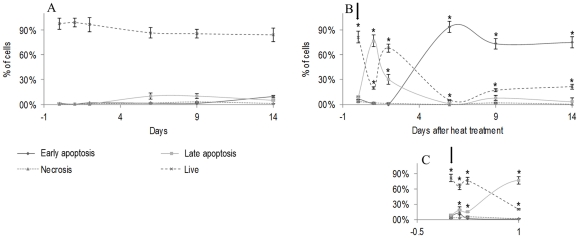
Apoptosis assessment of B16-F10 melanoma cells submitted to a heat treatment (HT), 45°C for 30 min. Flow cytometry analysis of Annexin-FITC/PI uptake to evaluate the percentage of cells in early and late apoptosis. Following the heat treatment, cultures were maintained at 37°C for 2 weeks. A: control cultures (kept at 37°C during the culture time); B: heat-treated cultures; C: detail of the heat-treated cultures during the first 24 h after heat exposure. *Significantly different from control (cultures kept at 37°C).

**Figure 4:**
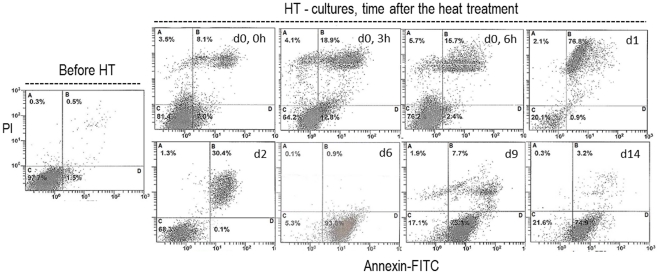
Apoptosis assessment of B16-F10 melanoma cells submitted to a heat treatment (HT), 45°C for 30 min. Representative histograms (from four independent experiments), before the heat treatment (d 0) and after, at defined time-points (d 0, 0 h–d 14, immediately to 14 days after heat exposure). Flow cytometry analysis of Annexin-FITC/PI uptake (A, necrosis; B, late apoptosis; C, viable cells; D, early apoptosis).

**Figure 5:**
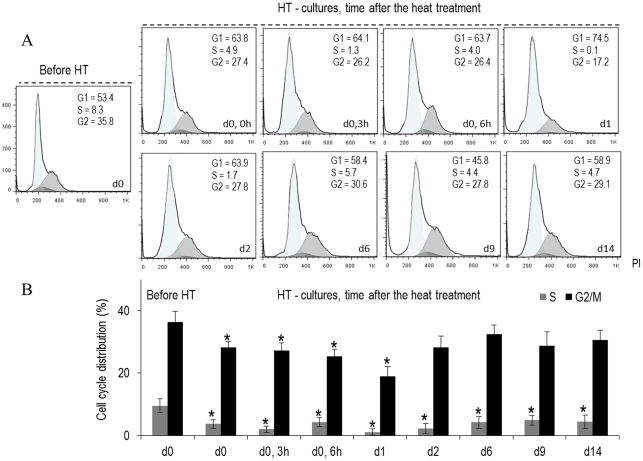
Cell cycle analysis of B16-F10 melanoma cells submitted to a heat treatment (HT), 45°C for 30 min, evaluated by flow cytometry. Representative histograms (A) (from four independent experiments) and quantitative data (B) of the control and the heat-treated cultures, at defined time-points throughout the culture period (d 0, 0 h–d 14, immediately to 14 days after heat exposure). *Significantly different from control (cultures kept at 37°C).

At defined time-points, the adherent cells present in control and heat-treated cultures were suspended and counted, and the results showed significantly lower values in the cultures submitted to hyperthermia, Table 2. Aliquots of these cell suspensions were analysed by flow cytometry for the presence of apoptosis and cell cycle phase, Figures 3, 4, 5.

**Table 2:** Number of adherent cells/cm^2^ in control and heat-treated cultures throughout the culture period, at defined time-points.

Days	0	0.125 (3h)	0.250 (6h)	1	2	6	9	14
Control	50.0×10^4^	49.3×10^4^	53.3×10^4^	56.8×10^4^	48.0×10^4^	48.0×10^4^	43.2×10^4^	38.7×10^4^
	(±4.6×10^4^)	(±4.4×10^4^)	(±4.8×10^4^)	(±5.1×10^4^)	(±4.3×10^4^)	(±4.4×10^4^)	(±3.8×10^4^)	(±3.4×10^4^)
Heat-treated Cultures	39.2×10^4^	5.2×10^4^	2.8×10^4^	2.4×10^4^	3.5×10^4^	1.9×10^4^	0.9×10^4^	1.1×10^4^
	(±3.3×10^4^)	(±0.4×10^4^)	(±0.2×10^4^)	(±0.3×10^4^)	(±0.2×10^4^)	(±0.2×10^4^)	(±0.1×10^4^)	(±0.1×10^4^)

Control cultures exhibited a high percentage of live cells throughout the culture time, Figure 3A. In the heat-treated cultures, Figure 3B, significant changes occurred during the first 24 h following the heat shock. The percentage of live cells decreased immediately after the treatment (∼64%, at 3 h), increased slightly in the next hours (∼75%, at 6 h), but at 24 h, the number of live cells was low (∼20%), Figures 3B, C. During this phase, cultures presented a high percentage of cells in late apoptosis. Following, there was a slight recovery, and 2 d after the heat treatment, the percentage of live cells was around 68%. At later times, the surviving cells were engaged in early apoptosis, i.e. 6 d after the heat shock, cultures presented ∼5% of live cells and ∼94% of early apoptotic cells, Figure 3B. In the following days, the percentage of live cells increased again, an average of 18–20% at 9 d and 14 d after the heat treatment. Figure 4 shows representative histograms of the cultures before and following the heat treatment.

Regarding cell cycle analyses, Figure 5 shows that at the culture time selected for the heat treatment, control cultures presented most of the cell population in G_1_ and G_2_/M phases, with a small percentage of cells in S phase. After the heat exposure, the percentage of S cells decreased significantly and was almost negligible 1 d and 2 d after the treatment; at this stage, the cell population remained mostly in G_1_ phase. Following, 6 d–14 d after heat exposure, there was a small increase in the percentage of S phase cells (∼5%) accompanied by a slight decrease in the percentage of G_1_ phase cells, and the culture remained stable during this period.

However, it is worth mentioning that the number of cells in heat-treated cultures was significantly lower than that in control cultures, as shown in Table 2.

### Gene expression profile

**Figure 6:**
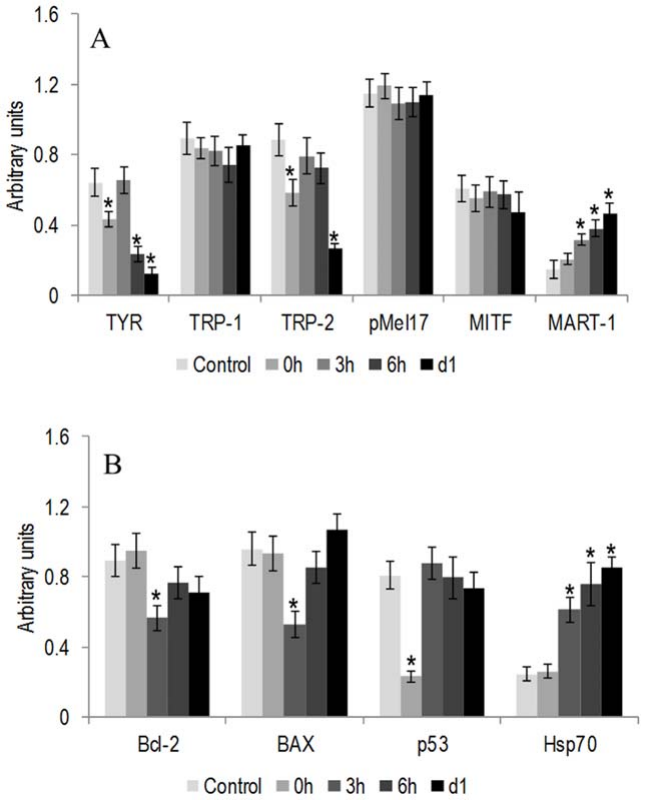
RT-PCR gene expression of B16-F10 melanoma cells submitted to a heat treatment (HT), 45°C for 30 min. Expression of TYR, TRP-1, TRP-2, pMel17, MITF and MART-1(A) and Bcl-2, BAX, p53 and Hsp70 (B) during the first 24 h following the heat treatment. RT-PCR products were subjected to a densitometric analysis and were normalized to the corresponding β-actin value. *Significantly different from control (cultures kept at 37°C).

Control cultures, at day 5 (the day of the heat treatment), expressed genes that are involved in melanin production, cell proliferation and differentiation events, namely TYR, TRP-1, TRP-2, pMel17, MITF and MART-1. Hyperthermia caused a significant decrease in the expression of TYR immediately after the heat treatment, a recovery after 3 h and a high decrease at 6 and 24 h. Expression of TRP-2 also decreased immediately after the heat shock, recovered at 3 and 6 h and decreased significantly at 24 h. Expression levels of pMel17 and MITF were similar to control, and MART-1 expression increased progressively after the heat treatment, attaining significantly higher values at 3, 6 and 24 h. Results are shown in Figure 6A.

At the same stage of the culture (day 5), control melanoma cells expressed genes associated with cell response to injury, Bcl-2, BAX, p53 and Hsp70. Hyperthermia induced some significant changes. Expression of Bcl-2 and BAX decreased 3 h after the heat treatment, but recovered afterwards, especially in the case of BAX. Expression levels of p53 suffered a significant decrease immediately after the heat treatment, recovered to normal values at 3 h, but, afterwards, decreased slowly (although without statistical significance). Expression of Hsp70 was similar to control immediately after the treatment, but increased significantly afterwards. Results are presented in Figure 6B.

## Discussion

Hyperthermia is a cancer therapy based on the knowledge that heat can kill cells after a temperature breakpoint, above fever temperature [1]. Although it's already being used as cancer therapy, there still is a lot to uncover regarding the effects that are critical to the destruction of cancer cells and cancerous tissues. An important feature of hyperthermia is the period of the heat shock reaction. Most studies focused in the immediate effects, and the long-term effects on the surviving cells are less known. In this work, the melanoma cell line B16-F10 was submitted to hyperthermia, at 45°C for 30 min, and cell response was characterized regarding the acute and long-term effects of the heat treatment.

Control cultures (maintained at 37°C) attained confluence around day 7, explaining the slow increase in the viability/proliferation observed afterwards. Accordingly, cell cycle analysis revealed that most of the cell population was in G_1_ phase. Towards later incubation times, cultures showed already a low percentage of late apoptotic cells, which is probably related with the aging of the confluent cell layer. The hyperthermia treatment induced significant acute and long-term effects in this behaviour.

Detailed characterization of the acute cell response to the heat treatment showed significant cell damage during the first 24 h, with a time-dependent cell response, which resulted in low number of adherent viable cells (Figure 1; Table 2). Cultures exhibited a disrupted cellular organization, an altered actin cytoskeleton and, at day 1, the remaining cells showed typical features of late apoptotic/necrotic cells (Figure 2). This was confirmed by the flow cytometry results (Figures 3 and 4). Also, cell cycle analysis revealed a negligible percentage of S phase cells (Figure 5). These observations suggest that in the acute heat shock reaction, cell death occurred mainly by a process of late apoptosis/necrosis, which would be expected from exposure to a severe trauma, such a high temperature [28]. In this drastic mechanism of cell death, the membrane-permeabilized cells release a variety of intracellular molecules that may damage neighbouring cells leading to later manifestations of cytotoxicity [28]. Following, assessment of the small remaining population throughout a period of 14 d after the heat exposure showed several discrete attempts of cell recovery from the inflicted damage. However, the number of adherent cells remained low until the end of the culture period, and, in this later stage, a high percentage of cells were in early apoptosis (Figures 3 and 4). During this early stage of programed cell death, dying cells are characterized as having an intact plasma membrane but expose PS on the cell surface, a process that, in physiological conditions, mediate their recognition by phagocytes and subsequent clearance [27], [28]. In this study, the presence of early apoptotic cells long after the heat shock might result from more subtle effects of the acute cell response which take longer to be expressed, and/or from indirect cytotoxicity induced by the release of toxic molecules from the high number of late apoptotic/necrotic cells generated during the acute phase reaction. However, nevertheless the significant cell injury elicited by the heat exposure, a small percentage of cells was able to survive long after the treatment. In line with this, cell-cycle analysis revealed that most of the remaining cells were in G_1_ phase, but cultures still maintained a low but relatively constant percentage of cells engaged in S and G_2_/M phases (Figure 5), suggesting the presence of a metabolically active cell population.

The *in vitro* effects of hyperthermia have been studied in several cell lines. Temperatures in the range of 45°C have induced alterations in a variety of cellular parameters [22]. Some cells, like RIF-1 line, appear to be resistant to this treatment and cellular viability was not affected [22]. Other cells, such as melanoma cells (Bowes) and lung adenocarcinoma cells, have demonstrated to be tolerant to hyperthermia for a short period, but afterwards cell viability decreased [19]. A great number of cell lines appears to present a low tolerance to hyperthermia at 45°C, showing a variety of deleterious effects including alterations in cell viability and increase of cellular death by apoptosis or necrosis, as observed in human erythroleukaemia cells [21], murine melanoma cells [23] and prostatic stromal cells [29]. The effect of hyperthermia in melanoma cells have been reported in a few studies, although with a great variability of experimental protocols, which difficult the comparison with the present study. In human melanoma cells heated from 41–43°C for 3 and 6 h, an increase on the shedding of soluble intercellular adhesion molecule-1 (sICAM-1) was found, with maximum values at 3 h [14]. Also, human melanoma cells were evaluated for cell survival and gene expression as a function of temperature (41.8–45°C) and time of exposure (15–150 min) for a period up to three days after the heat shock [15]. Another study showed that human melanoma cells incubated at 43°C for 120 min showed poor heat resistance and a higher fibrinolytic potential, measured immediately after the heat shock [19]. B16-F10 murine melanoma cells were also addressed in a study involving the treatment at 43 and 46°C in a PBS-EDTA solution for 30 min, and showed a significant decrease in cell viability immediately after hyperthermia [23]. Some of the deleterious substances that are expected to be generated as a result of the heat exposure are free radicals and reactive oxygen species (ROS). Flanagan and co-workers have described that treatment at 45°C can increase the flux of free radicals and ROS [30]. These are highly reactive species that can interact with key molecules, leading to cellular damage [31], [32]. Grasso et al, though at a lower temperature (42°C), also reported that the formation of ROS after hyperthermia contributes to growth arrest [33].

As described above, the heat treatment caused acute and long-term effects on the melanoma cell line B16-F10. However, the most significant effects were noted during the first 24 h after the heat shock, and the present results suggested that this acute response had a determinant effect in the long-term behaviour of melanoma cells, as cultures never recovered from the drastic reduction in the number of adherent/live cells that occurred during this period. Considering this, cultures were also assessed for the expression of melanoma functional genes and genes involved in response to stress, during the first 24 h of the heat shock reaction.

Control cultures, assessed at day 5 of the culture time (at the moment of the heat treatment), was in a pre-confluent stage, as referred above, and expressed several typical melanoma genes, namely TYR, TRP-1, TRP-2 and pMel17, genes that are involved in melanin synthesis. Melanin pigment protects the skin against UV-induced skin damage through its optical and chemical filtering properties, being essential for skin homeostasis. It is synthesized in the melanosomes, being the end-product of multistep transformations of L-tyrosine, under complex regulatory mechanisms [34], [35], [36]. Expression of MITF was also observed; this gene encodes a transcription factor that has a key role in melanoma cells by regulating cell cycle progression, survival, differentiation and invasion, and also modulates the expression of several pigmentation genes [36], [37]. MART-1, which function is not entirely understood although it appears to be involved in melanosome biogenesis [36], [38], was also expressed. The heat treatment decreased significantly the expression of TYR (and to a lesser extent, TRP-2), suggesting a deleterious effect in the expression of genes associated with the synthesis of melanin. Cultures were heated at day 5, at a pre-confluent stage, being greatly engaged in melanin synthesis, with might explain this deleterious effect. These results are in accordance with previous studies in human melanoma cells reporting a decrease in the expression of TYR in the first hours after heat-treatment at 45°C for 22 min [15]. The heat treatment had little effect in the expression of MITF, most probably because, as referred above, cultures were relatively stabilized regarding cell proliferation and cell cycle progression, which might explain the similar expression in control and heat-treated cultures. MITF appears to have a role in the expression of pMel17 [36], [37], which probably contributes for the lack of effect of the heat shock in the expression of this gene. On the other hand, expression of MART-1 was not affected in the first hours following the heat treatment, but an increase was observed afterwards, compared to control, probably reflecting an attempt to restore melanosome biogenesis. In a previous report using human melanoma cells MART-1 expression was not affected [15]. Although the temperature used was the same, 45°C, the heating time was only 22 min, which could explain the different results. An exposition to the heat for a longer period has been reported to increase cell damage [15], [17].

Control cultures also expressed genes associated with apoptotic events and cell response to stress, namely Bcl-2, BAX, p53 and Hsp70. Control cultures showed a similar expression profile of the competing genes of the same family Bcl-2 and BAX, respectively an anti-apoptotic gene and a pro-apoptotic gene [39], expression of p53, encoding a transcription factor that is activated as part of the cell response to stress that regulates many downstream target genes [40], and low levels of Hsp70, a gene with a key role in the repair mechanisms following cell injury [41]. The heat treatment induced some significant alteration in this gene profile. A similar decrease in the gene expression of the two competing genes Bcl-2 and BAX was observed 3 h following the heat shock, followed by a recovery to values similar to control. Regarding these two genes, it has been shown an increase in the expression of BAX and a decrease in Bcl-2 two hours after hyperthermia treatment at 42.5°C [42], [43], using HL60, HSB2 and Kasumi cell lines. This is in line with that occurring in physiological conditions due to the role of these genes [39]. In the present work, the use of a higher temperature, as 45°C, appears to create impairment in the behaviour of both genes probably due to a severe cytotoxic effect observed at this high temperature. Expression of p53 was greatly affected immediately after the heat shock, returned to normal values in the next few hours, however showing a tendency for a decrease, which might suggest a progressive impairment in coping with the inflicted cell injury. It is known that p53 protein has a key role in the heat shock reaction, acting as a transcription factor in cell cycle regulation, and loss of its function results in genetic instability and an impaired induction of apoptosis [40]. Regarding this, it appears that cell lines with lower levels of p53 protein have a decreased susceptibility to undergo apoptosis [20]. Hsp proteins can repair and restore the structures and functional integrity of damaged proteins, having a role in the protection against hyperthermia-induced apoptosis [40], [41], [44]. The present results showed that the expression of Hsp70 did not change immediately after the heat shock, but increased few hours later, attaining significantly higher values compared to control. This observation is in agreement with a previous report using human melanoma cell lines and the same temperature, with an augmentation in Hsp70 expression few hours after treatment [15]. Also, previous studies performed in hematopoietic and erythroleukaemia cells reported that, at 45°C, cells were able to induce Hsp70 only few hours after hyperthermia treatment [20], [21].

It is worth mentioning that, *in vitro*, some details in the experimental protocol might have a significant influence in the effects of the heat exposure. In this study, after the heat treatment, cells were kept for 48 hours in the same culture medium. Other authors have chosen to change the medium immediately after treatment [45]. Keeping the cells in the same medium after heat shock, would contribute to potentiate the harmful effects because of the longer contact time with toxic molecules liberated to the medium by damaged and dead cells.

### Conclusion

In conclusion, the highly metastatic melanoma cell line B16-F10 is susceptible to hyperthermia treatment, at 45°C for 30 minutes. Immediately after the heat shock, and in the first 24 h, cells began a process of fast degradation, with decreased viability, alterations in morphology and a significant reduction of the number of adherent cells, most of them in late apoptosis/necrosis, suggesting significant inflicted toxic effects. During the same period, the heat treatment also caused evident changes in the expression of functional melanoma cell genes, and in genes associated with cell response to injury. A follow-up of two weeks after heat exposure showed that viability and number of live cells remained very low and, after few days, cells were in early apoptosis. Still, it is worth mentioning that heat-treated cultures maintained a low but relatively constant percentage of cells in S and G_2_/M phases long after heat exposure, suggesting the presence of metabolically active cells.
